# Using Data for Good in New England, Mississippi, and 38 Cities in China: Building Age-Friendly Momentum

**DOI:** 10.1093/geroni/igaf122.349

**Published:** 2025-12-31

**Authors:** Elizabeth Dugan, James Connell

## Abstract

This symposium describes how researchers can collaborate with state leaders to transform data into tools that identify health inequities and support social change. The 2025 Healthy Aging Data Reports (see healthyagingdatareports.org) have been released in five New England states (Connecticut, Maine, Massachusetts, New Hampshire, and Rhode Island), Mississippi (2023), and Wyoming (2023). Related work is underway in 38 cities in China. The background, data sources, statistical methods, and findings are reviewed in Dugan’s opening overview presentation. Then state leaders describe efforts and progress in Maine, Massachusetts, Mississippi, New Hampshire, and Rhode Island. James Fuccione describes the Massachusetts Healthy Aging Collaborative work with policy leaders, philanthropy, academics, service providers, and private sector leaders. James Connell describes Age-Friendly Rhode Island efforts, including developing a strategic plan, convening high-level policy meetings, creating a website, and successfully advocating for resources. Jennifer Rablais describes the New Hampshire Alliance for Healthy Aging (NHAHA) efforts to develop a strategic plan, engage stakeholders to prioritize activities, and utilize a collective impact approach. Dr. Kina White describes the inspiring progress of age-friendly Mississippi which has convened briefings, trainings, and annual Age-Friendly Summits. Jess Mauer describes the Maine Council on Aging and the Power in Aging movement in Maine that aims to eliminate ageism by 2032. Finally, Dr. ShuangShuang Wang describes the development and use of a healthy aging index in China. Dr. Wang leads a panel discussion of best practices, lessons learned, and mistakes to avoid to help those in other places seeking to create age-friendly social change.

